# Deterioration of Kidney Function Is Affected by Central Arterial Stiffness in Late Life

**DOI:** 10.3390/jcm13051334

**Published:** 2024-02-27

**Authors:** Lisanne Tap, Kim Borsboom, Andrea Corsonello, Fabrizia Lattanzio, Francesco Mattace-Raso

**Affiliations:** 1 Section of Geriatric Medicine, Department of Internal Medicine, Erasmus MC, University Medical Center Rotterdam, 3000 CA Rotterdam, The Netherlands; 2 Italian National Research Center on Ageing (IRCCS INRCA), 60124 Ancona, Italy; 3 Department of Pharmacy, Health and Nutritional Sciences, University of Calabria, 87100 Cosenza, Italy

**Keywords:** hemodynamics, arterial stiffness, aortic pulse wave velocity, blood pressure, aging, chronic kidney disease, kidney function, cardiovascular disease

## Abstract

Cardiovascular diseases affect kidney function. The aim of this study was to investigate the possible associations between hemodynamic parameters and change in kidney function in individuals aged 75 years and older. Data on hemodynamics and blood and urine samples were collected at baseline and during one-year visits. Hemodynamics were split into two groups based on median values. Changes in the estimated glomerular filtration rate (eGFR) were investigated between low and high groups for each hemodynamic parameter using analysis of variance. Changes in the albumin–creatinine ratio (ACR) were examined as binary outcomes (large increase vs. stable) using logistic regression. The population consisted of 252 participants. Participants in the high central systolic blood pressure (cSBP) group had a greater decline in eGFR than participants in the low cSBP group (−6.3% vs. −2.7%, *p* = 0.006). Participants in the high aortic pulse wave velocity (aPWV) group had a greater decline in eGFR than those in the low aPWV group (−6.8% vs. −2.5%, *p* = 0.001). Other hemodynamic parameters were not associated with eGFR changes. Hemodynamics were not associated with changes in the ACR; aPWV and cSBP appear to be predictors for eGFR decline in older age; monitoring and treatment of elevated stiffness might be helpful in order to prevent kidney function decline.

## 1. Introduction

Chronic kidney disease (CKD) is a common finding in older individuals and is associated with increased risk of morbidity and mortality [1,2]. Individuals in late life, especially those with CKD, are more likely to develop cardiovascular diseases. Several risk factors are known to cause kidney function decline, such as aging, hypertension and diabetes mellitus [1]. In contrast to age, these cardiovascular risk factors are modifiable and therefore important factors in screening and treatment for CKD [2,3].

Relevant cardiovascular age-related changes are decline in cardiac output and stroke volume [4]. Aging also causes changes in vascular walls, which leads to a decrease in the elasticity of the vessels [5]. As a result, vascular resistance and arterial stiffness will increase. These age-related processes can be accelerated by different risk factors like hypertension, hypercholesterolemia and diabetes mellitus [6]. In addition, these age-related changes might also affect the kidney in several ways. Lower cardiac output and stroke volume can alter the renal blood flow, whereas vascular stiffening can expose the microcirculation to hemodynamic stress. Both processes could eventually lead to damage in the kidneys [7,8]. Also, kidney function decline can influence bone mineralization and promotes vascular calcification [9]. This makes cardiovascular diseases both a cause and consequence of kidney function decline.

The association between cardiovascular condition and CKD in older individuals has been investigated. However, the results are conflicting and the possible association, especially in the oldest populations, remains unclear [8,10,11,12,13,14]. The aim of this study was to investigate the effect of hemodynamic parameters on the changes in kidney function within one year in older individuals. We hypothesized that older individuals with poor hemodynamics at baseline would have a greater decline in kidney function during follow up than those with favorable hemodynamics.

## 2. Materials and Methods

### 2.1. Study Population

The present study was conducted within the framework of the “Screening for Chronic kidney disease among Older People across Europe” (SCOPE) study, an international, observational and prospective cohort study [15]. A detailed description of the study protocol can be found elsewhere [15]. A comprehensive geriatric assessment (CGA) was performed at baseline and during follow-up visits. Inclusion criteria of the SCOPE study were as follows: participants had to be 75 years or older and had to attend outpatient services or clinics. Patients with end-stage renal disease (ESRD; estimated Glomerular Filtration Rate (eGFR) < 15 mL/min/1.73 m^2^) or renal dialysis, end-stage heart failure (New York Heart Association Classification (NYHA) class IV), a history of solid organ or bone marrow transplantation, an active malignancy of metastatic cancer within 24 months prior to the visit, a life expectancy of less than 6 months, a severe cognitive impairment (Mini-Mental State Examination (MMSE) < 10/30) or patients unwilling to provide consent were ineligible for the study.

The subgroup used for the present study consisted of 301 participants enrolled in the Netherlands. In this subset, data from baseline and one-year follow-up visits were used. Participants who could not be followed up and whose hemodynamic or kidney function parameters could not be collected were excluded from the present study. The Medical Ethics Committee of the Erasmus Medical Center in Rotterdam reviewed and approved the SCOPE study. All participants provided written informed consent.

### 2.2. Hemodynamics and Kidney Function

Blood and urine samples were collected at baseline and after one year of follow up. The BIS 1 (Berlin Initiative study) equation was used to determine kidney function [16]. This creatinine-based formula is accurate for estimating the GFR of older individuals. Calculation of eGFR-BIS1 was performed by using serum creatinine, age and sex of the participants. Also, albuminuria was measured to assess kidney function. Consistent with previous literature, we defined a large increase in the urine Albumin-to-Creatinine Ratio (UACR) as a 2-fold increase in UACR during follow up, the development of a UACR ≥ 2.5 g/mol for men who had UACR < 2.5 at baseline or the development of a UACR ≥ 3.5 g/mol for women who had UACR < 3.5 at the first visit [11].

Hemodynamic parameters were determined with the Mobil-O-Graph (IEM, Rheinland, Germany) [17]. The Mobil-O-Graph is a non-invasive device for the analysis of peripheral systolic and diastolic blood pressure, mean arterial pressure, pulse pressure and heart rate [18,19]. The software of this device has the ability to analyze these measurements and is able to calculate central blood pressure, aortic pulse wave velocity (aPWV) and the augmentation index. With the software, other hemodynamic parameters were also assessed. These parameters included cardiac output, stroke volume, cardiac index and total peripheral resistance [20].

The parameters used in this study include the following: (central) systolic blood pressure (cSBP), in mmHg; (central) diastolic blood pressure (cDBP) in mmHg; stroke volume, in mL, cardiac output (CO), in L/min; cardiac index ^a^ (CI) in L/min/m^2^); total peripheral resistance (TPR) in s·mmHg/mL); central pulse pressure (cPP) in mmHg; aortic pulse wave velocity (aPWV) in m/s ^b^.

^a^ Cardiac index assesses cardiac output values based on body size. It is denoted in liters/minute/body surface area measured in meters squared.

^b^ Pulse wave velocity is the velocity of the pulse wave to travel between two points in the arterial system, measured in m/s.

### 2.3. Statistical Analyses

The Shapiro–Wilk normality test was conducted for characteristics and variables to test the normality of the data distribution. Categorical data were expressed in percent prevalence (%). Depending on the normality of the data distribution, continuous data were noted as means ± SD or median [IQR].

The parameters of interest were (c)SBP, (c)DBP, stroke volume, CO, CI, TPR, cPP and aPWV. Each parameter was split into two groups based on the median considering the relatively small group of participants included and the lack of specific reference values for multimorbid older adults. The group with outcomes equal to and below the median were called “low” and the group with outcomes above the median were called “high”. The baseline table is also stratified for low and high aPWV. Continuous variables were compared with an independent sample *t*-test or Mann–Whitney U test. Categorical variables were compared with the Chi-square test.

Change in eGFR (in percentage) within one year was investigated in low and high groups for each hemodynamic parameter using analysis of variance (ANOVA). Changes in UACR were examined as binary outcomes (large increase vs. stable) by performing logistic regression. If an association was found between a hemodynamic parameter and change in eGFR or a large increase in UACR, further multivariate analyses were performed using analysis of covariance (ANCOVA) or multivariate logistic regression per parameter. Potential confounders were identified and those with a *p*-value < 0.1 were used in adjusted analysis using different models, where appropriate. Age and sex were not included in the models, since comparable annual eGFR change can be expected for adults above the age of 75 years [21]. *p*-values < 0.05 were considered statistically significant. Statistical analyses were performed using IBM SPSS Statistics version 25 for Windows.

## 3. Results

In total, 301 participants were enrolled in the SCOPE study in the Netherlands. Eventually, 277 participants were successfully followed up for a period of one year. Patients with missing values were excluded. Therefore, the study population consisted of 252 participants.

### 3.1. Baseline Characteristics

The health-related baseline characteristics are presented in Table 1. The mean age of the participants was 80.1 ± 4.4 years old and 41.3% of the participants were women. Among all the participants, 71.8% used antihypertensive drugs. Hypertension was found in 176 participants (69.8%) and diabetes mellitus in 65 participants (25.8%). Participants in the high PWV group (aPWV > 12.2 m/s, n = 121) were older than those in the low group (83.0 vs. 76.9 years, *p*-value < 0.001). Participants in the low aPWV group (n = 131) used beta blockers (50.4% vs. 32.2%, *p* = 0.004) and statins more often than participants in the high aPWV group (57.3% vs. 34.7%, *p* < 0.001). Participants in the high aPWV group did not have higher prevalence of cardiovascular events, heart failure, atrial fibrillation or diabetes mellitus compared to the participants in the low aPWV group.

Table 2 describes the hemodynamic and laboratory characteristics at baseline stratified for aPWV. The mean aPWV in the total cohort was 12.4 ± 1.2 m/s. Participants in the high aPWV group in general had higher blood pressure values (both peripheral and central) than participants in the low aPWV group. For instance, the SBP was 158.0 vs. 141.0 mmHg (*p* < 0.001), MAP was 119.4 vs. 107.6 mmHg (*p* < 0.001) and cPP was 49.4 vs. 39.1 mmHg (*p* < 0.001), respectively. The TPR was higher in the high aPWV group than in the low aPWV group (median 1.5 vs. 1.3 s·mmHg/mL, *p* = 0.008). Stroke volume, CO and CI did not differ between groups. At baseline, the mean eGFR in the low aPWV group was 49.0 ± 13.6 mL/min and 46.2 ± 12.7 mL/min in the high aPWV group with no statistically significant difference between the groups. Participants in the high aPWV group had a higher UACR (median 3.4 vs. 1.9 g/mol, *p* = 0.008) than those in the low aPWV group.

### 3.2. Hemodynamic Parameters and Change in eGFR

Table 3 shows the difference in kidney function expressed in percentage change in eGFR within one year for the different hemodynamic parameters according to median values. Participants in the high cSBP group showed a greater decline in eGFR than the participants in the low cSBP group (−5.9% vs. −3.2%, *p* = 0.042). Likewise, participants in the high aPWV group had a greater decline in eGFR compared to those in the low group (−6.5% vs. −2.8%, *p* = 0.005). The change in eGFR for participants in the high groups of SBP, DBP, cDBP, stroke volume, CO, CI, TPR and cPP did not differ from those in the low groups of those hemodynamic variables.

**Figure 1:**
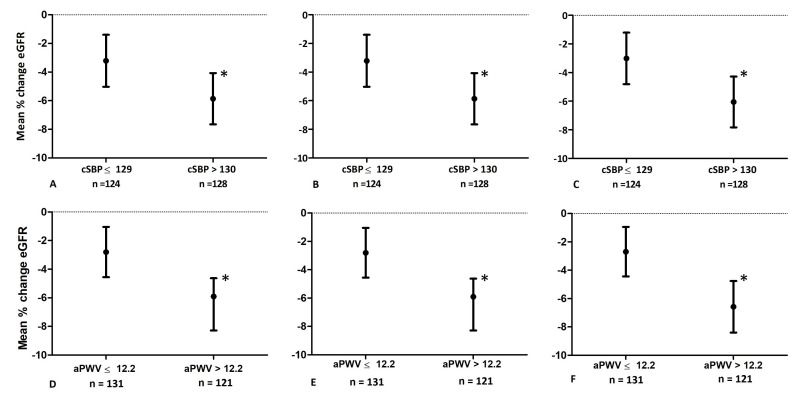
Association between baseline cSBP (panel **A**–**C**) or baseline aPWV (panel **D**–**F**) and percentage change in eGFR (n = 252): (**A**) adjusted for baseline eGFR; (**B**) adjusted for baseline eGFR, BMI, and DM; (**C**) adjusted for baseline eGFR, BMI, DM, the use of ACE inhibitors and beta blockers; (**D**) adjusted for baseline eGFR; (**E**) adjusted for baseline eGFR, BMI, DM, and hypertension; (**F**) adjusted for baseline eGFR, BMI, DM, hypertension, the use of ACE inhibitors and beta blockers. Dots represent means and bars represent 95% confidence interval. Significant differences between groups are marked with an asterisk (*). Abbreviations: aPWV, aortic pulse wave velocity; BMI, body mass index; cSBP, central systolic blood pressure; DM, diabetes mellitus; eGFR, estimated glomerular filtration rate.

Figure 1 shows the adjusted mean percentage change in eGFR in participants with low and high cSBP (panels A–C) and in those with low and high aPWV (panels D–F). In the adjusted analyses, participants in the high group of cSBP had a greater decline in eGFR than those in the low group. In the extensive adjusted model (panel C), the mean percentage change in eGFR was −2.7% in the low cSBP group and −6.3% in the high cSBP group (*p* = 0.006). Comparable results were found in the analysis for aPWV, where participants in the high group of aPWV had a greater decline in eGFR than participants in the low group of aPWV. In the extensive adjusted model (panel F), the mean percentage change in eGFR was −2.5% in the low aPWV group and −6.8% in the high aPWV group (*p* = 0.001)

### 3.3. Hemodynamic Parameters and Change in UACR

For all hemodynamic parameters, there were no differences between the low and high groups in the association between the baseline parameters and the chance of a large increase in the UACR. 

## 4. Discussion

In the present study in individuals aged 75 years and over, we found that participants in the highest categories of cSBP and aPWV had a greater decline in kidney function within one year than participants in the lowest category of cSBP and aPWV with an additional decline of 2.7% and 3.7% in one year, respectively. These results suggest that increased aortic stiffness has a negative effect on kidney function (even) in older age. All included hemodynamics appeared to have no influence on changes in UACR.

Several mechanisms can explain the found associations. Due to age-related changes in the vascular wall and the presence of additional cardiovascular risk factors, wall elasticity decreases and vascular resistance and arterial stiffness increases [5,6]. Also, elevated arterial stiffness is associated with hypertension [22,23]. Thus, with the stiffening of the arteries, an increase in (central) SBP and increase in aPWV can be observed. Previous studies showed that increased aortic stiffness is associated with changes in the microvascular structure in kidneys [8,11]. Moreover, due to vascular contraction, the arterial flow of the cortex could decrease, both potentially resulting in kidney function decline [8,24]. Increased central blood pressure, as well as increased pulsatility is associated with end-stage organ tissue damage [7], particularly in an autoregulated organ such as the kidney. Since central blood pressure is a direct reflection of the fluctuations in pressure that small arterial vessels face, high cSBP might result in impairment of kidney function [25]. It should be noted that age, blood pressure and aortic stiffness are all intertwined and related. Therefore, the found associations could be the result of the linked age-related microcirculatory changes, including loss of GFR and may not suggest causality.

Our hypothesis suggesting lower cardiac output, cardiac index and stroke volume would be associated with kidney function decline was not confirmed in the present study. As stiffening of the arteries, decline in cardiac output and stroke volume are also age-related changes [26,27], this might not affect kidney perfusion due to sufficient compensation mechanisms. In people with lower cardiac output or stroke volume, redistribution of blood volume in the body could prevent a decrease in blood flow to the kidneys to preserve kidney function [28].

Our finding that aPWV is associated with kidney function decline is in line with previous studies [8,11,12]. Due to different ways of determining kidney function, follow-up time and different age groups, the results could not be completely compared.

In contrast to our results, these previous studies also found an association between cPP as a marker of arterial stiffness and kidney function decline [8,11,12]. A large longitudinal retrospective registry-based cohort found that high-baseline cPP predicted kidney function decline in participants aged 60 to 79 years old, but not in participants above 80 years [29]. This is in line with the present study, with an average age of 80 years, in which we did not find an association between cPP and eGFR decline. Moreover, a community-based study with participants over 85 years old did not find an association between pulse pressure and decline in creatinine clearance [30]. Furthermore, aPWV is a more reliable marker for arterial stiffness than cPP as aPWV is based on arterial properties only, whereas cPP is based on arterial properties and cardiac function [31].

In the present study, pSBP and both cDBP and pDBP were not associated with changes in kidney function. Firstly, fluctuations in the central arteries are a reflection of the pressure that the vessels in kidneys are actually being exposed to [25], which could be an explanation of the found association in central measures but not in peripheral. Secondly, both SBP and DBP increase with age; however, over the age of 60 only SBP increases, whereas DBP remains stable (or even decreases) [32]. This could be another explanation as to why associations between DBP and kidney function were not found.

In the present study, we did not find associations between hemodynamics and changes in UACR. Consistent with our findings, a longitudinal study in participants in the same age group found that higher baseline aPWV and cPP were not associated with UACR increase, which were both analyzed as categorical and continuous variables [11]. Alternatively, in a cohort of the Framingham heart study, higher carotid–femoral PWV at baseline was associated with incident microalbuminuria over a 7- to 10-year period of follow up [33]. This association attenuated after adjustment for different risk factors. The differences in findings could be explained by the use of different cut-off values for albuminuria. Furthermore, differences between study populations, especially age and follow-up time, could account for the differences between previous studies and our results.

Several limitations need to be discussed. First, parameters were split into two groups based on the median considering the relatively small group of participants included and the lack of specific reference values for multimorbid older adults. The high PWV group is older, had higher SBP and PP and lower eGFR at baseline; Despite using multivariate models, intrinsic bias and confounding due to this factor may not be fully eliminated and have an impact on the found results. Second, the study has a relatively short follow-up-time of 1 year. However, despite this small sample size and short follow-up time, we were able to detect significant results. Larger studies with a longer follow-up time might be needed to investigate this topic further. Furthermore, we have focused on older individuals aged 75 years and older and the majority are Caucasian. Therefore, the generalizability could be limited. Survivor bias might also have played a role in attenuating the relationship between hemodynamics and kidney function. In addition, we used the UACR from a random portion of urine, which is a practical method to collect urine in older or frail adults. However, the collection of urine over 24 h is more reliable to investigate albuminuria. Moreover, the Kidney Disease Improving Global Outcomes (KDIGO) suggest that albuminuria could also be transient, suggesting a repeated measurement after three months to confirm this finding [34]. It should also be acknowledged that the Mobil-O-Graph calculates aPWV using an algorithm essentially based on age and blood pressure values, resulting in the interpretation of aPWV as a combined index of vascular aging [18,35]. It should be stated that the Mobil-O-graph is mainly validated for blood pressure and pulse wave analyses; therefore. analyses on other hemodynamics parameters could be less reliable. In addition, there may be evidence of confounding in the reported variables; for instance, the high aPWV group or high cSBP group might have had more nephrosclerosis before the start of the study and thus experience a greater decline in eGFR during the study as a result of this. Unfortunately, we have no information on the trajectories of the patients’ kidney function and concomitant pathology before inclusion in the study. Therefore, we adjusted for relevant identified covariates at baseline such as, among others, baseline eGFR, diabetes and hypertension. Finally, there were no restrictions in smoking, drinking coffee or tea or medication use on the day of the measurements, which might have influenced the measurements of arterial stiffness. Nonetheless, circumstances were comparable between the groups. Hence, this should not have affected the results. The present study also has several strengths. One of the strengths is the fact that the BIS 1 equation was used to determine kidney function, which is developed for older populations and gives a more accurate estimation of kidney function [16]. Moreover, a comprehensive geriatric assessment was performed, which is able to capture numerous domains of health status and their complex interactions in older and/or frail adults. To our best knowledge, this is the first study that has investigated various hemodynamics and their effect on kidney function in individuals aged 75 years and over.

## 5. Conclusions

In conclusion, we found that elevated central aortic stiffness is associated with a greater decline in kidney function in old age. Since aPWV and cSBP both appear to be predictors of eGFR decline, it might be of interest to identify older individuals with elevated aortic stiffness. In this specific population, intensive blood pressure reduction might be justified in order to slow down the process of vascular aging and prevent kidney function decline. It would be of interest to expand the study population to investigate the relationship between hemodynamics and kidney function decline in a large study population and longer follow-up time. Additional functional imaging techniques (sonography, computed tomography, magnetic resonance imaging or positron emission tomography) might also be of additional informative value to better understand the structural changes as a result of vascular and kidney aging.

## Figures and Tables

**Table 1:** Health-related baseline characteristics stratified for aPWV.

	All Participants (n = 252)	aPWV ≤ 12.2 m/s (n = 131)	aPWV > 12.2 m/s (n = 121)	*p* Value
Age, years	79.1 [76.6–83.0]	76.9 [75.5–78.4]	83.0 [79.6–85.9]	*p* < 0.001
Women, %	41.3	42.7	39.7	*p* = ns
BMI, kg/m^2^	26.2 (3.9)	26.4 (3.9)	26.1 (3.9)	*p* = ns
MMSE, score	29 [27–30]	29 [28–30]	28 [28–30]	*p* = ns
ADL independent, %	83.3	86.3	80.2	*p* = ns
iADL independent, %	47.2	58.0	35.5	*p* = ns
Alcohol				*p* = ns
no, (%)	64.3	64.1	64.5	
1–2 units/day, %	26.2	26.7	25.6	
>2 units/day, %	9.5	9.2	9.9	
Smoking				*p* = ns
No, %	29.8	32.1	27.3	
Former, %	65.1	62.6	67.8	
Current, %	9.5	5.3	5.0	
Medication				
Antihypertensives, %	71.8	74.0	69.4	*p* = ns
ACE inhibitor, %	19.8	17.6	22.3	*p* = ns
Beta blocker, %	41.7	50.4	32.2	*p* = 0.004
CA channel blocker, %	23.0	22.1	24.0	*p* = ns
Diuretics, %	19.8	21.4	18.2	*p* = ns
Statins, %	46.4	57.3	34.7	*p* < 0.001
Comorbidities				
Hypertension, %	69.8	64.1	76.0	*p* = 0.040
CVA, %	7.9	6.1	9.9	*p* = ns
TIA, %	14.7	13.0	16.5	*p* = ns
HF, %	12.7	13.0	12.4	*p* = ns
MI, %	17.9	18.3	17.4	*p* = ns
AF, %	17.1	17.6	16.5	*p* = ns
DM, %	25.8	29.8	21.5	*p* = ns
CIRS, severity index	1.8 (0.3)	1.8 (0.3)	1.8 (0.3)	*p* = ns
CIRS, total	12.7 (4.9)	12.8 (4.9)	12.5 (5.0)	*p* = ns

Abbreviations: ADL, activities of daily living; AF; atrial fibrillation; aPWV, aortic pulse wave velocity; BMI, body mass index; CA, calcium; CIRS, Cumulative Illness Rating Scale; CVA, cerebral vascular accident; DM, diabetes mellitus; HF, heart failure; iADL, instrumental activities of daily living; MI, myocardial Infarction; MMSE, Mini-Mental State Examination; TIA, transient ischemic attack. Note: non-normally continuous variables are presented as median [interquartile range] and normally distributed continuous are presented as mean ± SD. Categorical variables are presented as percentages. Continuous variables were compared between the PWV ≤ median and PWV > median with an independent sample *t*-test or Mann–Whitney U test. Categorical variables were compared with a Chi-square test. *p*-values < 0.05 were considered statistically significant.

**Table 2:** Hemodynamics and laboratory baseline characteristics stratified for aPWV.

	All Participants (n = 252)	aPWV ≤ 12.2 m/s (n = 131)	aPWV > 12.2 m/s (n =121)	*p* Value
Hemodynamics				
SBP, mmHg	147.0 [135.0–160.0]	141.0 [129.0–151.0]	158.0 [144.0–171.0]	*p* < 0.001
DBP, mmHg	86.2 (11.2)	83.3 (10.1)	89.4 (11.5)	*p* < 0.001
cSBP, mmHg	130.9 (17.5)	123.2 (12.9)	139.3 (18.0)	*p* < 0.001
cDBP, mmHg	86.8 (11.4)	84.0 (10.1)	89.9 (11.9)	*p* < 0.001
MAP, mmHg	113.3 (14.1)	107.6 (11.3)	119.4 (14.3)	*p* < 0.001
HR, beats/min	69.8 (12.1)	67.9 (11.3)	71.8 (12.7)	*p* = 0.014
Stroke volume, mL	75.3 (13.2)	76.5 (14.3)	74.0 (11.9)	*p* = ns
CO, L/min	5.2 (1.0)	5.1 (0.9)	5.3 (1.1)	*p* = ns
CI, L/min/m^2^	2.8 [2.3–3.2]	2.7 [2.3–3.2]	2.8 [2.4–3.3]	*p* = ns
TPR, s·mmHg/mL	1.3 [1.1–1.6]	1.3 [1.1–1.5]	1.5 [1.2–1.7]	*P* = 0.008
cPP, mmHg	44.0 (12.8)	39.1 (9.3)	49.4 (13.9)	*p* < 0.001
aPWV, m/s	12.4 (1.2)	11.6 (0.5)	13.4 (0.9)	*p* < 0.001
Kidney function				
eGFR, mL/min	47.6 (13.7)	49.0 (13.6)	46.2 (13.7)	*p* = ns
UACR, g/mol ^1^	2.7 [0.9–10.7]	1.9 [0.6–7.5]	3.4 [1.2–12.3]	*p* = 0.008

^1^ Missing data of 1 participant for PWV ≤ 12.2 m/s. Abbreviations: aPWV; aortic pulse wave velocity; cDBP, central diastolic blood pressure; CI, cardiac index; CO, cardiac output; cPP, central pulse pressure; cSBP, central systolic blood pressure; DBP, diastolic blood pressure; HR, heart rate; SBP, systolic blood pressure; TPR, total peripheral resistance; UACR; urinary albumin creatinine ratio. Note: non-normally continuous variables are presented as median [interquartile range] and normally distributed continuous are presented as mean ± SD. Categorical variables are presented as n (percentages). Continuous variables were compared between the PWV ≤ median and PWV > median with an independent sample *t*-test or Mann–Whitney U test. Categorical variables were compared with a Chi-square test. *p*-values < 0.05 were considered statistically significant.

**Table 3:** Mean change in eGFR within one year in low group (below median) and high group (above median) of different hemodynamics (n = 252).

Parameter (Median)	Low Group % Change in eGFR (95%CI)	High Group % Change in eGFR (95%CI)	Difference between Groups (95%CI)
SBP (147)	−3.6 (−5.6,−1.6)	−5.6 (−7.1, −3.9)	2.0 (−0.6, 4.5)
DBP (85)	−4.2 (−6.0, −2.4)	−5.0 (−6.8, −3.1)	0.8 (−1.8, 3.4)
cSBP (129)	−3.2 (−5.0, −1.4)	−5.9 (−7.7, −4.1)	2.7 (0.1, 5.2)
cDBP (85)	−4.5 (−6.3, −2.7)	−4.6 (−6.4, −2.8)	0.1 (−2.5, 2.7)
Stroke volume (74.6)	−4.0 (−5.8, −2.2)	−5.1 (−6.9, −3.3)	1.1 (−1.5, 3.7)
CO (5.1)	−3.8 (−5.6, −2.0)	−5.3 (−7.1, −3.5)	1.5 (−1.1, 4.1)
CI (2.8)	−4.1 (−6.0, −2.3)	−5.0 (−6.8, −3.2)	0.8 (−1.7, 3.4)
TVR (1.3)	−4.7 (−6.6, −2.7)	−4.5 (−6.2, −2.7)	−0.2 (−2.8, 2.4)
cPP (42)	−3.9 (−5.7, −2.1)	−5.3 (−7.1, −3.4)	1.4 (−1.2, 4.0)
aPWV (12.2)	−2.8 (−4.6, −1.0)	−6.5 (−8.3, −4.6)	3.7 (1.1, 6.2)

Abbreviations: aPWV, aortic pulse wave velocity; cDBP, central diastolic blood pressure; CI, cardiac index; CO, cardiac output; cSBP, central systolic blood pressure; cPP, central pulse pressure; DBP, diastolic blood pressure; SBP, systolic blood pressure; TPR, total peripheral resistance; Note: bold is *p* < 0.05.

## Data Availability

Data will be available for SCOPE consortium upon request from the principal investigator, Fabrizia Lattanzio, Italian National Research Center on Aging (IRCCS INRCA), Ancona, Fermo and Cosenza, Italy. f.lattanzio@inrca.it.
